# Differential disease resistance response in the barley necrotic mutant *nec1*

**DOI:** 10.1186/1471-2229-11-66

**Published:** 2011-04-15

**Authors:** Anete Keisa, Krista Kanberga-Silina, Ilva Nakurte, Laura Kunga, Nils Rostoks

**Affiliations:** 1 Faculty of Biology, University of Latvia, 4 Kronvalda Boulevard, Riga, LV-1586, Latvia

## Abstract

**Background:**

Although ion fluxes are considered to be an integral part of signal transduction during responses to pathogens, only a few ion channels are known to participate in the plant response to infection. CNGC4 is a disease resistance-related cyclic nucleotide-gated ion channel. *Arabidopsis thaliana *CNGC4 mutants *hlm1 *and *dnd2 *display an impaired hypersensitive response (HR), retarded growth, a constitutively active salicylic acid (SA)-mediated pathogenesis-related response and elevated resistance against bacterial pathogens. Barley CNGC4 shares 67% aa identity with AtCNGC4. The barley mutant *nec1 *comprising of a frame-shift mutation of CNGC4 displays a necrotic phenotype and constitutively over-expresses *PR-1*, yet it is not known what effect the *nec1 *mutation has on barley resistance against different types of pathogens.

**Results:**

*nec1 *mutant accumulated high amount of SA and hydrogen peroxide compared to parental cv. Parkland. Experiments investigating *nec1 *disease resistance demonstrated positive effect of *nec1 *mutation on non-host resistance against *Pseudomonas syringae *pv. *tomato *(*Pst*) at high inoculum density, whereas at normal *Pst *inoculum concentration *nec1 *resistance did not differ from wt. In contrast to augmented *P. syringae *resistance, penetration resistance against biotrophic fungus *Blumeria graminis *f. sp. *hordei *(*Bgh*), the causal agent of powdery mildew, was not altered in *nec1*. The *nec1 *mutant significantly over-expressed race non-specific *Bgh *resistance-related genes *BI-1 *and *MLO*. Induction of *BI-1 *and *MLO *suggested putative involvement of *nec1 *in race non-specific *Bgh *resistance, therefore the effect of *nec1*on *mlo-5*-mediated *Bgh *resistance was assessed. The *nec1*/*mlo-5 *double mutant was as resistant to *Bgh *as *Nec1*/*mlo-5 *plants, suggesting that *nec1 *did not impair *mlo-5 *race non-specific *Bgh *resistance.

**Conclusions:**

Together, the results suggest that *nec1 *mutation alters activation of systemic acquired resistance-related physiological markers and non-host resistance in barley, while not changing rapid localized response during compatible interaction with host pathogen. Increased resistance of *nec1 *against non-host pathogen *Pst *suggests that *nec1 *mutation may affect certain aspects of barley disease resistance, while it remains to be determined, if the effect on disease resistance is a direct response to changes in SA signaling.

## Background

To date, numerous lesion mimic mutants (LMM) have been characterized in *Arabidopsis thaliana*, rice and maize [1,2]. Frequently, LMM display enhanced disease resistance, constitutive expression of pathogenesis-related responses and an altered hypersensitive response (HR). Molecular mechanisms triggering the onset of cell death underlying the lesions mimic phenotype might have common features with HR-associated cell death observed during pathogen infection [3]. Although a direct link between HR and plant disease resistance is often questioned [3,4], it is evident that LMM can clarify numerous aspects of plant-pathogen interactions at the molecular level.

Although several barley mutants with necrotic leaf spots have been reported [5], only very few LMM phenotypes of barley have been traced down to a particular gene. The best known examples of barley LMM are *mlo *[6,7], and the recently characterized *necS1 *(*HvCAX1*) [8], which apart from displaying a necrotic phenotype also shows enhanced disease resistance against fungal pathogens. The barley mutant *nec1 *comprising of a mutated cyclic nucleotide gated ion channel 4 (CNGC4) exhibits the necrotic phenotype and over-expresses the pathogenesis-related gene *PR-1 *[9]. *A. thaliana CNGC4 *mutants *dnd2 *and *hlm1 *which are orthologous to barley *nec1 *mutants display enhanced resistance to virulent bacterial pathogens [10,11]. HvCNGC4 shares 67% aa identity with AtCNGC4 [9], suggesting that a similarly to *dnd2 *in *A. thaliana nec1 *mutation may affect barley disease resistance.

Bacterial diseases of barley have been described, although the mechanisms of resistance have not been studied in detail [12,13]. Apparently, there is no race-specific resistance to bacterial pathogens: thus, only PAMP-triggered immunity is operational, even though cultivar-dependent differences in infection rates have been reported for bacterial kernel blight caused by *Pseudomonas syringae *[14]. Significant over-production of salicylic acid (SA) upon *P. syringae *infection in barley suggests that barley resistance to non-host bacterial pathogens is achieved through a SA-mediated defense pathway [15].

Bacterial pathogens of *Arabidopsis *are commonly used as a model system for plant-pathogen interaction studies. However, fungal pathogens are the causal agents of economically more deleterious and widespread diseases in barley. Powdery mildew is caused by the biotrophic fungus *Blumeria graminis *f. sp. *hordei *(*Bgh*). This is among the best studied barley diseases, and extensive details are available on both the race specific or race non-specific powdery mildew resistance mechanisms [16]. Race non-specific resistance of barley to *Bgh *is a cell wall-based resistance forbidding fungal penetration into a host cell [17]. Penetration resistance is triggered by the ROR2 protein, presumably directing secretion vesicle trafficking to the fungal penetration site [18]. Race non-specific penetration resistance is fully attained only in the absence of the trans-membrane protein MLO which is a negative regulator of ROR2 [19]. Functional MLO protein employs Ca^2+ ^and CaM signaling to ensure fungal penetration into host cells. Mutations negatively affecting MLO binding with CaM render barley more resistant against *Bgh *[19,20], while overexpression of another trans-membrane protein, BI-1, counteract *mlo*-triggered *Bgh *resistance in a Ca^2+ ^and CaM signaling-dependent manner [21,22]. Although the interdependence of Ca^2+^/CaM signaling and race non-specific *Bgh *resistance in barley is well established, so far no Ca^2+ ^permeable ion-channel has been shown to participate in *Bgh *resistance or susceptibility.

Race specific resistance of barley against *Bgh *requires the presence of plant *R*-genes called *Ml *genes. In contrast to race non-specific *Bgh *resistance, race specific resistance usually permits fungal penetration into the host cell, but restricts further spread of the fungus by triggering plant cell death [16]. Both types of powdery mildew resistance have been shown to incorporate reactive oxygen species (ROS) signaling elements, such as increased accumulation of H_2_O_2 _and/or superoxide [23,24]. H_2_O_2 _acts as a principal signaling molecule initiating cell death during incompatible race-specific barley-*Bgh *interaction [24]. Early accumulation of H_2_O_2 _in mesophyll cells underlying attacked epidermal cells is proposed to be critical for the establishment of race specific resistance [25,26]. In race non-specific interactions, H_2_O_2 _plays a distinct role from that observed for HR induction. In *mlo*-triggered resistance, H_2_O_2 _most likely ensures host cell wall fortification, thus preventing fungal penetration [23,27].

In this study, disease resistance of barley LMM *nec1 *mutants displaying necrotic leaf spots was analyzed. Although *NEC1 *has been shown to encode cyclic nucleotide gated ion channel 4 (CNGC4) and to over-express the defense-related *PR-1 *gene [9], the effect of *nec1 *mutation on barley disease resistance has not yet been characterized. This study shows that *nec1 *mutation triggers the induction of H_2_O_2 _and SA, restricts *Bgh *microcolony formation and affects non-host resistance against *Pseudomonas syringae *applied at high inoculum density, whereas it has no effect on *Bgh *penetration efficiency or *mlo*-dependent race non-specific *Bgh *resistance.

## Results

### *nec1 *mutant exhibits constitutive activation of H_2_O_2 _and salicylic acid

The *nec1 *allele in cultivar Parkland was initially described as a natural mutation [28], which was confirmed by identification of a MITE insertion in an intron of the *NEC1 *gene that caused alternative splicing and a predicted non-functional protein [9]. The *nec1 *mutant line GSHO 1284 and a parental variety Parkland were genotyped with DArT markers [29]. Only 2.2% of 1131 DArT loci were polymorphic, suggesting that the mutant is essentially isogenic to Parkland (data not shown). All described experiments were performed with Parkland and its mutant *nec1 *accession GSHO 1284.

As it was found that *nec1 *significantly over-expressed pathogenesis related genes [9], it was investigated whether *nec1 *plants spontaneously display also other SAR-related signals such as altered accumulation of reactive oxygen species and over-accumulation of SA Spectrofluorimetric analysis of whole-leaf extracts of two week old *nec1 *plants with a fully developed lesion mimic phenotype and the parental line Parkland showed a three-fold higher overall level of H_2_O_2 _in the mutant (data not shown).

**Figure 1:**
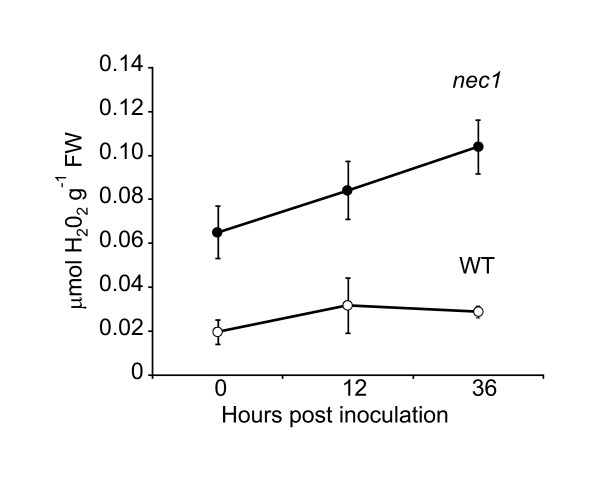
**Time course of whole leaf H_2_O_2 _accumulation in *nec1 *and wt plants after *Bgh *infection**. *nec1 *mutation triggers H_2_O_2 _over-accumulation in barley in the absence of pathogen infection, but it does not alter time course of H_2_O_2 _production in response to *Bgh *infection. Error bars represent the standard deviation of means (n = 5 per data point).

To ascertain whether the elevated overall amount of H_2_O_2 _in *nec1 *plants affected H_2_O_2 _accumulation during *Bgh *infection, overall H_2_O_2 _amount in *nec1 *and wt plants was assessed at 12 h and 36 h after inoculation with a virulent mixed population of *Bgh*. The analysis did not reveal considerable changes in the H_2_O_2 _content of wt plants during the first 36 h after inoculation, whereas *nec1 *mutants showed a slight, statistically non-significant increase in H_2_O_2 _levels at 36 h after inoculation (Figure 1).

**Figure 2:**
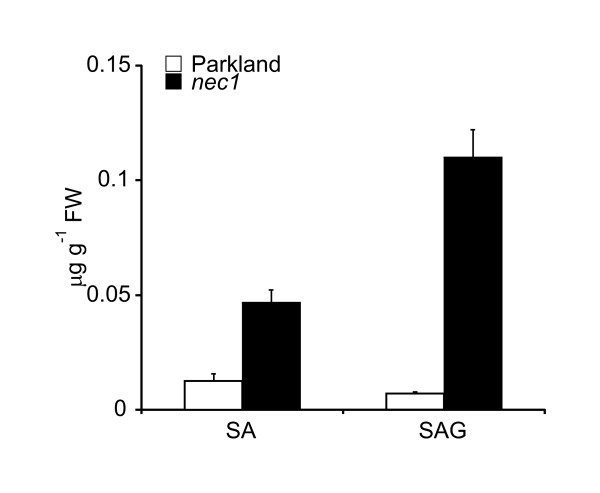
**Level of free and conjugated SA in *nec1 *and wt plants**. *nec1 *mutant contains significantly higher level of conjugated, as well as free SA compared to parental cv. Parkland. SA content was analyzed using reverse-phase high performance liquid chromatography in leaf tissue extracts of 14 day old plants. Average values from three biological replicates are presented, each consisting of three technical replicates. Error bars represent standard deviation.

H_2_O_2 _accumulation and *PR-1 *expression is known to be associated with SA-dependent signaling. Therefore, the SA content of *nec1 *and wt plants was also measured. HPLC assay confirmed that levels of free SA and conjugated SA were four- and fifteen-fold higher, respectively, in *nec1 *than in wt plants (Figure 2).

### Resistance of the *nec1 *mutant to *Pseudomonas syringae*

**Figure 3:**
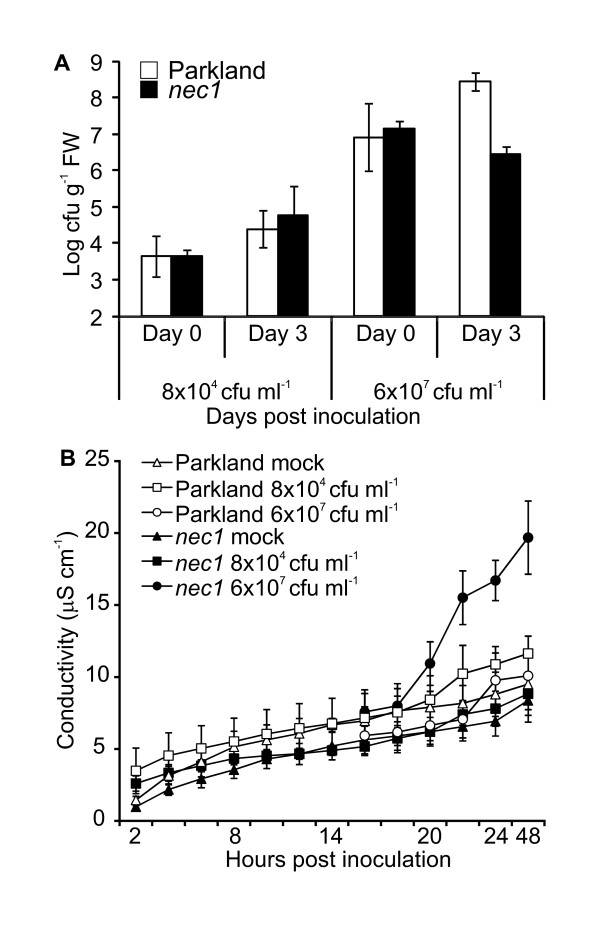
**Response of *nec1 *to non-host pathogen *Pseudomonas syringae *pv. *tomato *applied at low and high inoculum densities**. Panel A. Growth of *Pseudomonas syringae *pv. *tomato *in *nec1 *and parental cv. Parkland was monitored immediately and 3 days after vacuum infiltration with *Pst *applied at inoculum densities of 8 × 10^4 ^or 6 × 10^7 ^cfu ml^-1^. For mock inoculation plants were infiltrated with 10 mM MgCl_2_. Infection was expressed as number of colony forming units (cfu) per gram of fresh leaves (FW). Due to the high between-experiment variation, results of one representative experiment out of four independent experiments are shown. Error bars represent standard deviation. At high inoculum density (6 × 10^7^cfu ml^-^1) bacterial cfu number in *nec1 *at the day 3 was significantly (p < 0.01, Student's *t*-test) lower than in wt. Panel B. Progression of cell death in *nec1 *and Parkland after infection with *Pseudomonas syringae *pv. *tomato *in the experiment shown in panel A. *nec1 *mutation showed increased electrolyte leakage in barley inoculated with non-host bacteria *Pst *at 6 × 10^7 ^cfu ml^-1^. Measurements of electrolyte leakage were taken every two hours during 24 hour period and at 48 hours after inoculation. Error bars represent standard deviation.

Barley resistance to the non-host bacterial pathogen *Pseudomonas syringae *likely employs SA-mediated defense pathway [15]. Therefore, the constitutive activation of SA signaling in *nec1 *might contribute to its non-host resistance. *nec1 *plants were inoculated with *P. syringae *pv. *tomato *(*Pst*) at two inoculum densities - 8 × 10^4 ^and 6 × 10^7 ^cfu ml^-1 ^using vacuum infiltration technique. At day 3 after infiltration with 6 × 10^7 ^cfu ml^-1 ^of *Pst *the amount of bacteria in *nec1 *was reduced, whereas Parkland had accumulated ca. 6-fold higher amount of *Pst *making the difference in bacterial growth between wt and *nec1 *statistically highly significant (p = 0.01, Student's *t*-test) at this stage of infection (Figure 3A). Inoculation with *Pst *at lower inoculum density (8 × 10^4 ^cfu ml^-1^) did not reveal any differences in resistance between *nec1 *and wt plants (Figure 3A).

Ion leakage measurements were also performed to characterize the effect of *Pst *infection on *nec1 *and Parkland. Vacuum infiltration with *Pst *at lower inoculum density (8 × 10^4 ^cfu ml^-1^) did not elicit cell death in either *nec1 *or Parkland (Figure 3B). In contrast to inoculation with lower *Pst *density, inoculation with *Pst *at 6 × 10^7 ^cfu ml^-1 ^elicited differential response in *nec1 *and wt. Tissue samples from *nec1 *plants inoculated with *Pst *at 6 × 10^7 ^cfu ml^-1 ^displayed more pronounced ion leakage suggesting an increased cell death in *nec1 *after infection (Figure 3B).

### Resistance of *nec1 *mutant to powdery mildew *Blumeria graminis *f.sp. *hordei*

**Figure 4:**
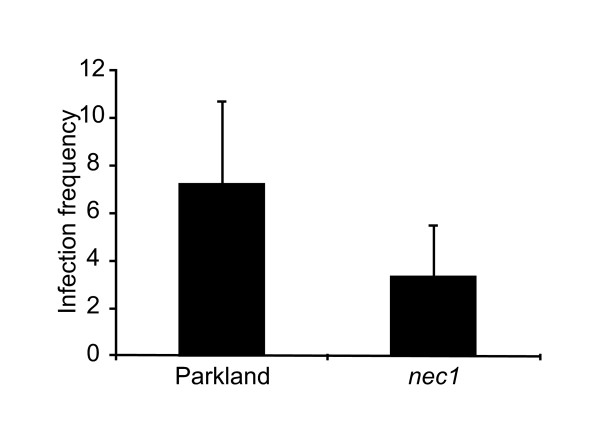
***Bgh *microcolony formation on *nec1 *and wt plants**. Excised segments of barley leafs were inoculated with a virulent *Bgh *isolate. Microcolony formation was inspected microscopically 4 days post infection and infection rate was expressed as a number of microcolonies per cm^-2 ^leaf area. Figure reflects data from two independent experiments. Error bars represent standard deviation. Infection frequency significantly differs between *nec1 *and Parkland (p < 0.001, *t*-test).

Since *nec1 *plants exhibited constitutively active defense responses, the role of *nec1 *in basal resistance against *Bgh *was assessed. Due to their basal resistance, even susceptible barley cultivars are able to restrict infection to some extent. In order to assess the effect of *nec1 *mutation on basal *Bgh *resistance, microcolony formation was examined. *nec1 *supported formation of significantly (p < 0.001, *t*-test) smaller number of *Bgh *colonies compared to wt plants (Figure 4). To further test, if restricted formation of *Bgh *microcolonies on *nec1 *derived from the rapid and effective localized response precluding fungal penetration or from post-invasive defense impeding further fungal development, we examined *nec1 Bgh *penetration resistance. The effect of *nec1 *mutation on *Bgh *penetration resistance was characterized as the proportion of interaction sites that had formed *Bgh *haustoria to the total number of *Bgh *spores that had germinated at 48 hpi. *nec1 *plants permitted almost identical entry and haustoria establishment rate of *Bgh *as the parental line (71% and 74% *Bgh *penetration efficiency respectively, p = 0.64, Student's *t*-test).

**Figure 5:**
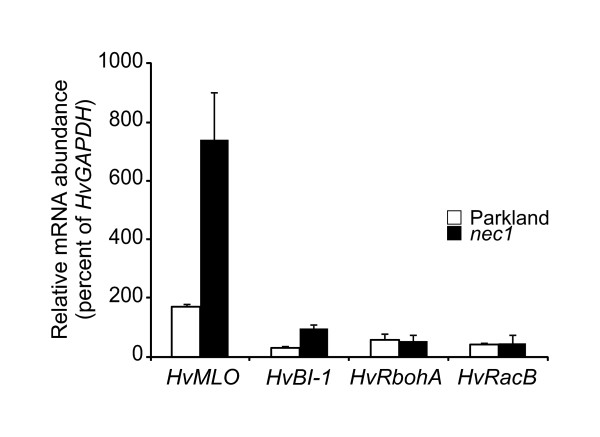
**Effect of *nec1 *mutation on expression of powdery mildew resistance related genes**. Transcript abundance of powdery mildew resistance related genes in *nec1 *mutants was determined by quantitative real time PCR. mRNA abundance of *HvMLO *and *HvBI-1 *is significantly increased in *nec1*. Error bars represent standard deviation.

Basal *Bgh *resistance has been shown to be tightly linked to the molecular mechanisms of race-specific *Bgh *resistance triggered by different *Mla *alleles [30,31]. *HvRbohA *and *HvRacB *are known to participate in basal as well as race-specific *Bgh *resistance [32-34]. The expression of these genes was characterized using real-time quantitative PCR. Relative mRNA abundance of the analyzed genes was not affected by *nec1 *mutation (Figure 5) indirectly suggesting that *nec1 *may be independent from effector-triggered immunity that ensure rapid localized *Bgh *resistance.

### *nec1 *mutation alters expression of *BI-1 *and *MLO*, but does not affect *mlo-5-*triggered race non-specific powdery mildew resistance

**Figure 6:**
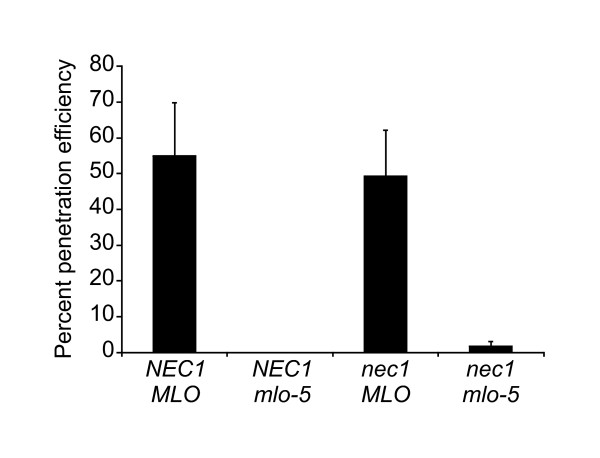
**Effects of *nec1 *mutation on *mlo-5 *triggered *Bgh *penetration resistance**. Fourteen days old plants were inoculated with 10-20 conidia per mm^2 ^and at 48 h post inoculation infected leaves were harvested and *Bgh *penetration efficiency was assessed. At least 100 interaction sites per variant were observed. Error bars represent standard deviation.

**Figure 7:**
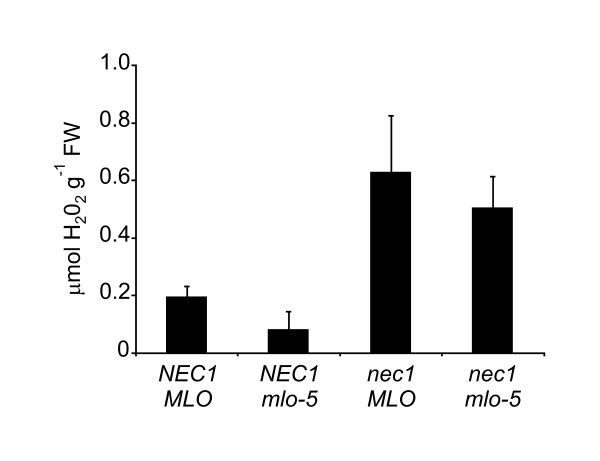
**Effect of *mlo-5 *mutation on H_2_O_2 _accumulation in barley mutant *nec1***. *mlo-5 *mutation does not affect over-accumulation of H_2_O_2 _in *nec1 *mutant. H_2_O_2 _content was determined spectrofluorimetrically in leaf extracts of wt, *nec1*, *mlo-5 *and *nec1*/*mlo-5 *double mutants. Error bars represent standard deviation.

Different powdery mildew resistance types employ at least partially distinctive molecular pathways: thus, a particular gene can have a significant role in one *Bgh *resistance strategy, while having only a marginal or no effect on another *Bgh *resistance type [35]. To find out, if *nec1 *mutation affected *mlo*-triggered race non-specific *Bgh *resistance, the expression of *MLO *and *BI-1 *genes was analyzed using real-time quantitative PCR. Loss of functional MLO protein renders barley almost fully resistant against *Bgh*, whereas *BI-1 *over-expression in *mlo *mutants leads to restoration of susceptibility against *Bgh *[22] and, in fact, *BI-1 *is required for full susceptibility of barley to powdery mildew [36]. Furthermore, over-expression of *MLO *in wild type plants leads to super susceptibility against *Bgh *[20]. Significant over-expression of both *MLO *and *BI-1 *in *nec1 *plants was observed (Figure 5). To further test whether *nec1 *mutation had any effect on race non-specific powdery mildew resistance conferred by *mlo-5 *mutation, *Bgh *penetration resistance of *nec1*/*mlo-5 *double mutant was characterized. Similar to *mlo-5 *mutant, *nec1*/*mlo-5 *plants were almost fully resistant to *Bgh*, allowing establishment of fungal haustoria only at less than 2% of interaction sites (Figure 6). In addition, the H_2_O_2 _content of whole-leaf extracts from *nec1*/*mlo-5 *double mutants was analyzed. While the *nec1 *mutant showed markedly increased accumulation of H_2_O_2 _compared to wt *NEC1 *plants, the experiment did not reveal a significant effect of *mlo-5 *mutation on H_2_O_2 _over-accumulation in *nec1 *(Figure 7).

## Discussion

Despite the fact that ion fluxes are known to play an important role in early signaling events during plant-pathogen interaction [37-39], to date only several plant ion channels have been shown to participate in plant disease resistance or plant-pathogen interaction signal transduction. The cyclic nucleotide gated ion channel (CNGC) gene family is one of the best-represented among the disease resistance-related ion channels. CNGC mutants *dnd1 *(AtCNGC2), *dnd2 *and *hlm1 *(AtCNGC4) and *cpr22 *(AtCNGC11/12) exhibit a wide range of pathogen resistance [10,11,40,41]. Mutations affecting AtCNGC4 enhance resistance of *Arabidopsis thaliana *against certain pathotypes of *Pseudomonas syringae a*nd *Botrytis cinerea *[10,11,42]. Although the effect of CNGC mutations on resistance against bacterial and oomycete pathogens is well-studied in *Arabidopsis*, little is known about the role of these genes in non-host resistance and also about the functions of CNGCs in disease resistance of economically important monocot plant species such as barley. Here we show that similarly to *dnd2 *in *A. thaliana *[10], *nec1 *in barley activates constitutive over-accumulation of SA. High level of SA contributes to enhanced disease resistance of *dnd2 *to virulent *Pseudomonas syringae *pv. *tomato *[10,42] and this resistance requires functional *PAD4 *[43], which is one of the central genes in SA-mediated effector-triggered immunity (ETI) [44] and SAR [45]. Although disease resistance pathways seem to be largely conserved among monocots and dicots [46-49], the position of SA in monocot immunity is ambiguous. Some monocots, such as rice, contain high endogenous SA levels [50] and SA is not required for *PR*-gene induction in rice upon infection [51]. Ineffectiveness of externally applied SA on induction of *PR*-genes has also been observed in barley [15] and wheat [52], however, inoculation with non-host bacteria *Pseudomonas syringae *triggers SA accumulation in barley [15]. Taking into account that such differences occur in the SA mediated resistance signaling among monocots and dicots, it is interesting to see whether mutation affecting SA mediated disease resistance in *A. thaliana *is also involved in barley disease resistance. The present study analyzed the effect of the *nec1 *(*HvCNGC4*) mutation on barley resistance against *Pseudomonas syringae *pv. *tomato *and *Blumeria graminis *f. sp. *hordei*.

Mutation in the *NEC1 *gene affected barley non-host resistance against *Pseudomonas syringae *pv. *tomato*. Bacterial growth in *nec1 *plants was delayed at the initial phase of infection, if plants were inoculated with bacteria at high inoculum density. At the same time the increased electrolyte leakage suggested somewhat enhanced cell death, even though the conductivity values were much lower than reported for typical HR. Thus, electrolyte leakage data in *nec1 *were generally in agreement with the expected "defense, no death" phenotype characteristic of *hlm1*/*dnd2 *mutants, although differences between *nec1 *and *hlm1*/*dnd2 *mutants may exist in this respect. Non-host resistance is predicted to share common defense responses with host resistance - either basal (PAMP-triggered immunity, PTI) or ETI [53,54]. The choice of which layer of immunity is activated upon a particular interaction with non-host pathogen seems to be case specific [55-57]. Therefore molecular mechanisms leading to changes in non-host resistance of *nec1 *to *P. syringae *pv. *tomato *might have also had an effect on interaction with host pathogens. This prompted the assessment of the role of *nec1 *mutation in resistance to powdery mildew caused by the fungal pathogen *Blumeria graminis *f. sp. *hordei*. *nec1 *restricted *Bgh *microcolony formation, while not affecting *Bgh *penetration or *mlo-5 *triggered resistance to *Bgh*. Interestingly, despite the fact that *nec1 *did not impede *mlo-5 *mediated race non specific resistance to *Bgh*, *MLO *and *BI-1 *mRNA abundance was significantly increased in barley *nec1 *plants (Figure 5). Significant over-expression of *MLO *and *BI-1 *might result from general activation of cell death-related signaling pathways and systemic immunity responses rather than from activation of particular powdery mildew resistance. Together these observations suggest that *nec1 *mutation most likely affects PTI and non-host resistance related responses and it is not associated with rapid localized defense responses required to prevent fungal penetration.

HR related cell death is suggested to serve in plant immunity as a factor triggering activation of SAR [4,58]. Spontaneous cell death might elicit constitutive activation of SAR related signaling pathway in *nec1*. Previously *nec1 *has been shown to constitutively over-express *PR-1a *and *β-1,3-glucanase *[9] - molecular markers of SAR. This study confirmed the constitutive activation of SA-related signaling pathways in *nec1 *mutants, since significant over-accumulation of H_2_O_2 _and SA in *nec1 *plants was detected. In *Arabidopsis thaliana*, non-host resistance against some types of pathogens involves SA signaling [59-61]. In barley, a substantial increase in SA levels has been observed after infection with *Pseudomonas syringae *pv. *syringae*, but not after inoculation with non-host fungus *Blumeria *(*Erysiphe*) *graminis *f. sp. *tritici *[15] or host pathogen *Bgh *[23] suggesting a differential role of SA in barley resistance against different pathogens. Constitutive activation of the SA-related defense pathway may contribute to differential resistance of *nec1 *mutant against non-host bacteria *Pst *and virulent host pathogen *Bgh*. However, the cause for SA over-accumulation needs further investigation, and it remains to be determined, if SA-independent pathways are activated in *nec1 *mutant similarly to *Arabidopsis hlm1*/*dnd2 *mutant.

## Conclusions

*nec1 *mutation increased resistance against the non-host bacterial pathogen *Pseudomonas syringae *pv. *tomato *applied at high inoculum density and it also inhibited microcolony formation of host pathogen *Blumeria graminis *f.sp. *hordei*, but its penetration resistance to *Bgh *or race non-specific *Bgh *resistance pathways were not impaired. The differential disease resistance response of *nec1 *plants might result from the activation of specific resistance pathways differentiating between various types of pathogens. SA-dependent signaling pathways have previously been shown to participate in disease resistance against certain types of pathogens, while not affecting others. *nec1 *mutant displays constitutive activation of systemic acquired resistance-related signals such as over-accumulation of hydrogen peroxide and SA, as well as over-expression of *PR-1*. It remains to be determined, if constitutive activation of SA related signaling is the main reason for the differential disease resistance of *nec1 *mutant.

## Methods

### Plants

Plants for all experiments were grown in an environmental growth chamber at 22°C under long-day (16 h day, 8 h night), medium light (ca. 150 μmol m^-2 ^s^-1^) conditions. The barley necrotic mutant *nec1 *(GSHO 1284) containing a MITE insertion in the gene for C*yclic Nucleotide Gated Ion Channel 4 *(*CNGC4*) [9] has previously been described as a natural mutant in cv. Parkland [28]. Both cv. Parkland and GSHO 1284 are completely susceptible to powdery mildew. *mlo-5 *and *nec1 *double mutant was obtained by crossing accession GSHO 1284 with NGB 9276 carrying the *mlo-5 *allele in the cv. Carlsberg II background [62]. Plants homozygous for *nec1 *and *mlo-5 *alleles were confirmed by genotyping the respective mutations and F_4 _plants were used for all experiments. Barley accessions GSHO 1284 and Parkland were obtained from USDA ARS National Small Grains Germplasm Research Facility (Aberdeen, Idaho, USA), and NGB 9276 was obtained from Nordic Genetic Resources Center (Alnarp, Sweden).

### Infection with *Pseudomonas syringae *pv. *tomato*

To study *nec1 *non-host resistance against *Pseudomonas syringae *pv. *tomato*, leaves of 14 day old *nec1 *plants were vacuum infiltrated with a bacterial suspension in 10 mM MgCl_2_. Bacterial suspension was applied at normal concentration 8 × 10^4 ^and high concentration 6 × 10^7 ^cfu ml^-1^, since low concentration inoculum typically applied for infection of host plants can have minor or no effect on non-host species [63]. For mock inoculation 10 mM MgCl_2 _was used for infiltration. Immediately after infiltration plants were covered with plastic bags to maintain high humidity and kept in dark for 1 h. After an hour plants were transferred to growth conditions described above. Bacterial growth was monitored at day 3 post inoculation by dilution plating of homogenized plant tissue. Leaves were briefly sterilized with 70% ethanol before homogenization. *Pseudomonas syringae *pv. *tomato *was obtained from the German microbial type collection (accession 50315).

### Cell death measurements

Cell death was quantified by electrolyte leakage assay performed as described by Dellagi et al. (1998) with minor modifications [64]. In brief, plants were vacuum infiltrated with *Pst *as described above and incubated in dark at high humidity for an hour. Five mm leaf disks were collected and washed with distilled water for 1 h and then transferred to a tube with 6.5 ml distilled water. Conductivity was measured with conductivity meter handylab LF11 (Schott Instruments). Each sample contained 4 leaf disks from 4 plants and at each data point 4 independent replicates were measured.

### Fungal material, inoculation and calculation of penetration efficiency

Two week old plants of *nec1 *and cv. Parkland were inoculated with 10-20 conidia per mm^2 ^from virulent mixed population of powdery mildew multiplied on cv. Parkland. For the characterization of penetration efficiency, infected barley leaves were harvested 48 h post inoculation and cleared for 24 h in 98% ethanol. Penetration efficiency was calculated as a ratio of interaction sites with haustoria formation and the total number of spores with developed appresoria. The overall penetration efficiency for the particular barley line was an average from three replicates containing at least 100 interaction sites each.

*Bgh *microcolony formation was examined on 5 cm long leaf middle segments, which were laid flat on 0.5% agar in water (w v^-1^) plates with adaxial surface facing up and were inoculated with mixed population of powdery mildew multiplied on cv. Parkland. Each plate contained leaves from both *nec1 *and cv. Parkland plants to compensate for uneven inoculation. *Bgh *microcolonies were microscopically scored 4 days post inoculation. Experiment was repeated twice with 14 independent samples per barley line in each experiment.

### H_2_O_2 _detection and quantification

Hydrogen peroxide was quantified spectrofluorometrically [65]. Briefly, 1 g of freshly harvested leaves from two week old barley plants were frozen in liquid nitrogen and ground in 50 mM Hepes-KOH buffer containing 1 mM EDTA and 5 mM MgCl2 (pH 7.5). After centrifugation for 10 minutes at 13000 g, the supernatant was transferred to a new centrifuge tube and an equal volume of chloroform:methanol (volume ratio 2:1) solution was added. After centrifugation for 3 minutes at 13000 g, the upper aqueous phase was transferred to a new centrifuge tube and 50 mM Hepes-KOH buffer solution (pH 7.5) containing 0.5 mM homovanillic acid and 15 U horseradish peroxidase VI was added to a final volume of 3 ml. Samples were incubated at room temperature for 30 minutes before fluorescence measurements were taken (excitation at 315 nm, emission at 425 nm). Fluorescence was measured with a FloroMax3 spectrofluorometer (Horiba Scientific, Japan). For quantification of the H_2_O_2 _a standard curve with a range of 100 μM - 1 nM was applied. Sample correction for quenching was performed by adding a known sample amount to a 10 nM H_2_O_2 _solution.

### Quantification of free and conjugated salicylic acid

The SA content in leaf tissue extracts was analyzed using reverse-phase high performance liquid chromatography (HPLC). Each sample contained leaf tissue from 3 two week old plants. Samples were prepared essentially as described [66]. Briefly, 0.45 g barley leaf tissue was homogenized in liquid nitrogen and sequentially extracted using 90% and 100% methanol. Extraction was repeated twice and two supernatant fractions were pooled and dried. The residue was resuspended in 1 ml of 5% acetic acid. As an internal standard for SA recovery correction, samples were selectively spiked with 50 μg per g FW 3-hydroxy benzoic acid (3-HBA) [66].

For the quantification of free SA, 1 ml of ethylacetate:cyclopentane:isopropanol (50:50:1) was added. The sample was thoroughly mixed and the upper phase (approximately 1 ml) was transferred to a new 2 ml tube. The aqueous phase was then re-extracted, as described previously, and both organic phases (approximately 2 ml) were pooled. The resulting solution was vacuum-dried and thoroughly resuspended in 0.9 ml of mobile phase. This suspension was filtered through a 0.20 μm filter.

The aqueous phase containing the SAG fraction was acidified with HCl to pH 1.0 and boiled for 30 min to separate free SA from conjugated SA. The released SA was then extracted with the organic mixture and treated as above.

Chromatographic analysis was performed on a modular HPLC system, Agilent 1100 series, consisting of quaternary pump, autosampler, column thermostat and both UV and fluorescence detectors (Agilent Technologies, Germany). Separation was achieved on a Zorbax Eclipse XDB-C18 (Agilent Technologies, Germany) column 4.6 × 250 mm, 5 μm. Column temperature was maintained at 40°C. The mobile phase was prepared by mixing acetonitrile:20 mM NaH_2_PO_4 _(pH 3.0 with acetic acid), in a volume ratio 25:75. The mobile phase flow rate was 1.0 ml min^-1^. Injection volume was 100 μl. The UV/VIS detector was set to 237 nm and 303 nm and the fluorescence detector to an excitation wavelength of 297 nm and an emission wavelength of 407 nm. Results were evaluated by a ChemStation Plus (Agilent, Germany).

### RNA extraction

For RNA extraction, 5 cm long segments of cotyledon leaf from two week old plants of necrotic mutant *nec1 *and parental cv. Parkland were frozen in liquid nitrogen immediately after harvesting. Total RNA was extracted from frozen leaf tissues using Trizol reagent. Each RNA sample was extracted from a pool of five plants, and three biological replicates of each barley line (15 plants in total) were used for expression analysis of *BI-1*, *MLO*, *HvRACB *and *HvRbohA *genes in *nec1 *and cv. Parkland plants. Integrity of the extracted RNA was monitored using non-denaturing agarose gel electrophoresis. Quantity of purified total RNA was monitored using spectrophotometer NanoDrop ND-1000 (NanoDrop products, USA). One to two μg of the extracted RNA was treated with DNaseI (Fermentas, Vilnius, Lithuania) following the manufacturer's instructions and afterwards purified using chloroform-ethanol extraction.

### Reverse transcription and quantitative real-time PCR

cDNA was synthesized with oligo (dT)_18 _primers in a total volume of 10 μl containing 1 μg of total RNA using the RevertAid H Minus First Strand cDNA synthesis kit (Fermentas, Vilnius, Lithuania).

For quantitative real-time PCR, aliquots of cDNA were amplified on an ABI Prism 7300 instrument (Applied Biosystems, Foster City, CA, USA) using the Maxima SYBR Green PCR kit (Fermentas, Vilnius, Lithuania) in a total volume of 20 μl containing 2 μl of cDNA and 0.3 μM primers (Table 1). The reaction was carried out as follows: initial denaturing step for 15 min at 95°C followed by 35 cycles of 15 s at 94°C, 30 s at 60°C and 45 s at 72°C (data acquisition step). Standard curves for the quantification of the transcript levels were calculated from serial dilutions of appropriate cDNA fragments amplified from cv. Parkland. Transcript levels of the studied genes were expressed as a percentage of *HvGAPDH *transcript value in the same sample. Combined values of two technical replicates of the three biological replicates (n = 6) were used to calculate the average values and standard deviations. Analysis of variance (ANOVA) of transcript abundance between the mutant and the corresponding parent was done in Microsoft Excel (Redmond, WA, USA).

**Table 1:** Quantitative real-time PCR primer sequences used in the study

Primer	Sequence	Reference
HvBI_cw1	CGATGATCTCCTGCGTGTCG	This study *
HvBI_ccw1	TACCTCGGTGGCCTGCTCTC	This study *
HvGAPDH_cw1	CGTTCATCACCACCGACTAC	[67]
HvGAPDH_ccw1	CAGCCTTGTCCTTGTCAGTG	[67]
MLO_F1	GTCGAGCCCAGCAACAAGTTCTTC	This study *
MLO_R1	ACCACCACCTTCATGATGCTCAG	This study *
HvrbohA_F1	CCGATCAGATGTATGCTCCA	[33]
HvrbohA_R1	CAGAAGGCATTGAAGCCAGT	[33]
HvRACB_L01	GGTAGACAAAGAACAAGGGCGAAGT	This study *
HvRACB_R01	CACAAGGCAGGAAGAAGAGAAATCA	This study *

* Primers were designed using Primer 3 software [68] using the following gene sequences as a template: *HvBI *(HarvEST21 Unigene 3323; AJ290421); *MLO *(HarvEST21 Unigene 6351; Z83834); *HvRacB *(HarvEST21 Unigene 5202; AJ344223)

## Authors' contributions

AK designed and performed the study and drafted the manuscript. KKS and LK performed the disease resistance tests and gene expression analyses. IN performed HPLC analysis and helped to draft the manuscript. NR designed and performed the study and wrote the final manuscript. All authors have read and approved the submitted manuscript.
